# To Biopsy a Small Renal Mass, or Not?

**DOI:** 10.5812/numonthly.3991

**Published:** 2012-03-01

**Authors:** Elefterios Chatzidarellis, Andreas Skolarikos, Athanasios G Papatsoris

**Affiliations:** 1 Department of Urology, School of Medicine, University of Athens, Sismanoglio Hospital, Athens, Greece

**Keywords:** Biopsy, Kidney

The abundant use of cross-sectional imaging has markedly increased the diagnosis of small renal masses (SRMs) (1). A strict definition of small renal masses is lacking and most authors consider lesions < 4cm as small (2). According to the literature, 20% of SRMs are benign in formal histological results and SRMs represent 48-66% of diagnosed renal cell cancers (RCC) (1, 2). In a meta-analysis by Chawla et al. (3) the incidence of metastasis of SRMs is 1% after a 3-year follow up. Nevertheless, the radiological features of SRMs are insufficient to describe the biological potential and predict the natural history of SRMs (4). Therefore, studies have demonstrated overtreatment of patients with benign SRMs which were misdiagnosed as malignant on the preoperative imaging (2, 4). The inability of modern imaging techniques (i.e. computerized tomography (CT) to differentiate benign from malignant SRMs has renewed interest in renal mass biopsy (RMB). Furthermore, the renewed interest in RMB is closely associated with novel minimal invasive treatments for SRMs (i.e. cryotherapy and radiofrequency ablation (RFA), improved biopsy techniques and pathological evaluation (5).

RMB aims to determine eventual malignancy, type and grade of the evaluated SRM with high specificity and sensitivity for the presence of malignancy. The diagnostic accuracy of RMB has improved through the last years, as has been demonstrated in a recent meta-analysis by lane et al. (5) The authors compared the diagnostic accuracy of RMB with reports stratified pre- and post-2001, showing an improvement from 88% to 94%. The diagnostic value of RMB refers not only to the differential diagnosis of benign versus malignant tumors, but also to the definition of the histological subtype of the tumor (i.e. renal cell carcinoma (RCC). Neuzillet et al. (6) showed high concordance (more than 91%), between the histological subtype findings of the RMB and the final nephrectomy specimen for SRMs (8).

The recent evolution of minimally invasive treatment options for SRMs highlights even more the important role of the RMB, before cryotherapy or RFA where no biopsy is possible (5). CT guided RMB has been demonstrated to improve the differential diagnosis of SRMs and prevent overtreatment (7). Furthermore, RMB can nowadays change the management of SRM which was doubtful a few years ago (8). A benign or SRM of low malignant potential can be managed with active surveillance protocols or ablative techniques especially in elderly and unfit patients. Moreover, the positive predictive value of imaging findings is so high that a negative RMB does not alter management (5). Furthermore, RMB is indicated in metastatic patients, before starting systemic therapy (7). In recent studies, RMB-related complications such as gross hematuria, renal hematoma requiring intervention (i.e. admission, transfusion or nephrectomy), arterio-venous fistula or pneumothorax are extremely rare (< 1%) (9).

The traditional concern of needle tract tumor seeding is based on only six reports published before 1993 (5). The majority of these cases were transitional cell carcinoma of the upper urinary tract, which is a contraindication for RMB. Interestingly, since 1993 no case of tumor seeding has been reported, most probably because of the innovative needle introducers which isolate the sample from the surrounding tissues.

Repeat RMB is suggested in cases of a non-diagnostic result due to an inadequate or insufficient specimen (9). In order to reduce the incidence of such non-diagnostic RMB there are several recommendations. RMB guided by CT and/or real time ultrasound increases diagnostic accuracy (10). Two cores of 15-22mm in length each taken by an 18-gauge biopsy gun provide reliable specimens (11). Furthermore, by targeting the peripheral area of the SRM, potential central necrosis is avoided. Moreover, by placing the tip of the needle a few millimeters outside the SRM, the specimen includes the capsule of the tumor. New molecular techniques such as, polymerase chain reaction and fluorescence in situ hybridization, improve the accuracy of tumor sub-typing (12). Moreover, modern immunocytochemistry can contribute to the differential diagnosis between RCC and several benign tumors, such as oncocytomas (13).

Nowadays, RBM has become an established tool for the management of SRMs as indicated in the recent European Association of Urology (EAU) guidelines (14). It is recommended for the diagnosis, follow-up surveillance and ablative therapies of SRMs. The future looks promising for RMB in the evaluation of SRMs, especially with the implications of molecular and cytogenetic profiling of the specimen. Eventually, RMB might be able to predict the prognosis of the SRM and guide its treatment. There is no easy answer to the difficult question, to biopsy a SRM or not, however the trend will be to continue to perform the biopsy.
